# Awareness and knowledge of dental erosion among Yemeni dental professionals and students

**DOI:** 10.1186/s12903-015-0103-x

**Published:** 2015-10-08

**Authors:** Amin Al-Ashtal, Anders Johansson, Ridwaan Omar, Ann-Katrin Johansson

**Affiliations:** Department of Clinical Dentistry - Cariology, Faculty of Medicine and Dentistry, University of Bergen, Årstadveien 19, PO Box 7804, 5009 Bergen, Norway; Department of Clinical Dentistry - Prosthodontics, Faculty of Medicine and Dentistry, University of Bergen, 5009 Bergen, Norway; Department of Restorative Sciences, Faculty of Dentistry, Kuwait University, Safat, 13110 Kuwait

**Keywords:** Awareness, Dental erosion, Dental students, Dentists, Knowledge, Yemen

## Abstract

**Background:**

This study explored Yemeni dentists’ and dental students’ knowledge about the associated factors, approaches to diagnosis and preventive methods of dental erosion (DE), as well as any associations between DE awareness and some potentially related variables.

**Methods:**

A self-administered questionnaire was distributed to general dental practitioners (*n* = 323) in Sanaa and to fifth year dental students (*n* = 97) at the University of Science and Technology, Sanaa, Yemen during the period from July to November 2013. Descriptive and logistic regression analyses were conducted.

**Results:**

Overall response rate was 79 %. Results indicated that 61 % of respondents learned about DE from dental school, 27 % from their own studies and the rest from continuing education courses and the media. DE was reportedly most frequently seen on incisors by 46 % of respondents and on premolars and molars by 24 %. While 48 % reported DE to be more common in male patients, no gender differences were reported by 20 % of respondents. Acidic drinks were believed to be causative factors for DE by 41 % of dentists and 52 % of students, while 90 % of respondents believed that patients’ dietary history is important during DE diagnosis. As preventive measures for DE, reduction of acidic drink consumption was advised by 51 % of respondents while only 37 % advised their patients to use fluoridated toothpastes. Younger respondents (≤35 years) were more likely to identify the commonly-known causative factors for DE (*P* = 0.024). Twenty-seven percent of the respondents reportedly used an index to grade DE. Dental students were more likely than dentists to use such an index (*P* <0.001) and to more frequently advise their patients to reduce intake of acidic drinks (*P = 0.02*) compared to dentists.

**Conclusions:**

In-depth knowledge about causative factors, diagnosis and preventive methods of DE was apparent among only half the respondents and approaches to early diagnosis were insufficient. There would appear to be an urgent need for enhancing awareness and knowledge about DE within the Yemeni dental community.

**Electronic supplementary material:**

The online version of this article (doi:10.1186/s12903-015-0103-x) contains supplementary material, which is available to authorized users.

## Background

Dental erosion has gained considerable attention from researchers during the last two decades [1, 2]. A high, but often varying, prevalence of dental erosive lesions has been reported especially in young populations [3–9]. Today, it is well-established that dental erosion is an oral health problem with a multifactorial background [1, 10]. It is important that dental professionals are able to diagnose the condition as early as possible, to identify the possible etiology of the erosive damage and also to understand the specific host defense factors of importance in each case. All this requires a careful and systematic evaluation of each patient by the dental professional, and particularly one who has a sufficiently good knowledge about tooth wear in general and about erosion specifically. In this regard, reports from Brazil and the United Kingdom showed that awareness of dental erosion among dental professionals was inadequate [11, 12].

Clinical diagnosis of dental erosion is considered difficult for dental practitioners [13–15]. This is further complicated by the fact that the condition is not well-known in the community [16] and most patients do not seek treatment for erosive lesions until the condition is at an advanced stage, when symptoms such as hypersensitivity or a perceived need for restorative therapy prompt the patient to seek treatment [17]. At the same time, there is clear evidence that an erosive process on a tooth will continue if the actual risk factors/behaviors persist and adequate preventive measures are not carried out [18]. Therefore, it is important to identify patients with dental erosion as early as possible. The diagnosis of dental erosion should also include the grading of its severity and if possible an assessment of its progression based on an evaluation of risk factors and behaviors present in each patient [18].

Early diagnosis of dental erosion depends mainly on the ability of the clinician to detect its pathognomonic features in enamel. However, a study conducted in a Brazilian dental school in 2011 showed that knowledge about dental erosion was not widely evident among students, patients and faculty members [12]. Of the 300 participants enrolled in the study, about a quarter of faculty members and over 60 % of dental students considered themselves unprepared to diagnose dental erosion. In other studies, patients were only occasionally or rarely advised by their dentists about any presence of dental erosion [11, 19].

For the previous reasons and since no information on the subject is currently available about Yemeni dental professionals, this study aimed to explore awareness and knowledge about dental erosion among a group of Yemeni dentists and dental students, and to assess associations between dental erosion awareness and some potentially related variables.

## Methods

This cross-sectional study was carried out in Sanaa, the capital of Yemen, with a population of nearly two million.

### Sample

Contact information of all members of the Yemeni Dental Association based in Sanaa was obtained from the Association, which included dental practitioners working in the government sector (*n* = 86) and in private dental clinics (*n* = 237). Another list of all enrolled dental students (*n* = 97) in year five (final year) was obtained from the Dental College administration at the University of Science and Technology (UST). These comprised a total of 420 and were all invited to participate in the study during the period from July to November 2013.

### Questionnaire

The questionnaire was developed in English (Additional file 1). It comprised four parts and contained 20 close-ended questions. The first part inquired about demographic information including sex, age, nationality, work sector, and for dentists, their date and place of graduation. The second part included questions about participants’ knowledge about dental erosion including its common clinical features and distribution among males and females. The third part enquired about dentists’ knowledge about dental erosion diagnosis, perceived etiological factors, and its clinical features. The last part of the questionnaire explored the preferred and applied dental erosion preventive methods used by participants.

The questionnaire was personally distributed to and collected from all participants by the principal investigator (AA). The aim of the study was explained to all invited participants and they were informed that participation in the study was voluntary and that return of the questionnaire was considered acceptance to participate. Participants who failed to return the questionnaire received two phone reminders after two and four weeks before they were reported as non-respondents.

### Pilot study

Prior to the start of the study, the questionnaire was piloted with ten Yemeni dentists and modified as needed.

### Statistical analysis

Data were analyzed using SPSS version 20.0 (IBM Corporation, Armonk, NY, USA). Response rates and gender distribution were calculated. Descriptive statistics were conducted to assess frequencies of answers in each part of the questionnaire relating to the level of awareness and knowledge of dental erosion. Logistic regression analysis was performed to examine possible associations between answers to questions on acidic drinks, systemic diseases and dental erosion preventive methods (dependent variables) and selected independent variables that included sex, age, nationality, country of graduation, type of participant (dentist or student), and referrals of erosion cases. Unadjusted (crude) odds ratios were calculated by conducting a separate logistic regression analysis for each selected variable. Then, a logistic regression analysis model was constructed by including all selected variables in the model to obtain adjusted odds ratios. Logistic regression models were constructed first for the whole sample, then for dentists and students separately. The results of possible associations were presented as OR (95 % CI) and *P* values. Statistical significance was set at *P* <0.05.

### Ethical considerations

The study protocol was approved by the Regional Committee for Medical and Health Research Ethics, Western Norway (Ref. 2013/981/REK Vest) and the Research Ethical Committee at the Faculty of Dentistry, University of Science and Technology in Yemen (Ref. 10/2013). Participation in the study was voluntary and without any compensation to respondents. Verbal consent was obtained from all participants. In addition, participants were informed that filling and return of the questionnaire would be considered as confirmation of acceptance to participate in the study. To ensure respondents’ anonymity, the questionnaire was number coded and identification information such as participant’s name or address was not required.

## Results

### Sample and respondents’ distribution

Of the total of 420 dentists and dental students invited to participate in the study, 332 agreed to do so and returned their completed questionnaires, giving an overall response rate of 79.0 % (332/420), while 72 dentists and 16 dental students did not return the questionnaire and were considered non-respondents. Group response rates were 77.7 % (251/323) among dentists and 83.6 % (81/97) among dental students. Respondents comprised 75.6 % (251/332) dentists and 24.4 % (81/332) dental students with age range between 21 and 58 years. Sample distribution and demographic data of respondents are shown in Table 1.

**Table 1:** Distribution and demographic data of respondents

Type of participant	N	Males	Females	Mean age	SD
Dentists	251	134 (53.4 %)	117 (46.6 %)	31.2	5.5
Dental students	81	38 (46.9 %)	43 (53.1 %)	23.7	2.3
Total	332	172 (51.8 %)	160 (48.2 %)	29.3	5.9

### Awareness and knowledge regarding dental erosion

More than half the respondents (60.5 %, 201/332) reported that they learned about dental erosion from dental school, 26.5 % (88/332) from their own studies and the rest from continuing education courses and the media (Table 2). According to participants’ opinions, dental erosion cases were seen more commonly in male patients by 48.2 % (160/332) of respondents and in female patients by 29.2 % (97/332), while the rest of the participants reported no gender differences among their patients.

**Table 2:** Distribution of respondents according to their stated source(s) of knowledge about dental erosion

Source of knowledge	Dentists	Students	Total
Dental school	154 (61.4 %)	47 (58.0 %)	201 (60.5 %)
Continuing education	10 (4.0 %)	6 (7.4 %)	16 (4.8 %)
Own studies	63 (25.1 %)	25 (30.9 %)	88 (26.5 %)
Media	0 (0.0 %)	1 (1.2 %)	1 (0.3 %)
Not answered	24 (9.6 %)	2 (2.5 %)	26 (7.8 %)
Total	251 (100 %)	81 (100 %)	332 (100 %)

Regarding the location of dental erosion, lesions were believed to be seen most frequently on buccal surfaces of teeth by 51.5 % (171/332) of respondents, on palatal surfaces by 16.3 % (54/332), and equally on all tooth surfaces by the remaining respondents. In terms of tooth type, 46.1 % (153/332) of participants reported that erosive lesions are most frequently found on incisors, 23.8 % (79/332) on premolars and molars, 20.2 % (67/332) equally on all teeth, while 9.9 % (33/332) did not answer the question.

### Knowledge regarding dental erosion causative factors

Forty-one percent (103/251) of dentists and 51.9 % (42/81) of dental students stated that acidic drinks are causative factors for dental erosion. On the other hand, 11.6 % (29/251) of dentists and 16.0 % (13/81) of dental students believed that bruxism and excessive tooth brushing with a hard toothbrush can cause dental erosion. Adjusted logistic regression analysis showed that younger respondents (≤35 years) were more likely to regard acidic drinks as causative factors for dental erosion than older respondents (OR = 3.18; *P* = 0.009) (Table 3).

**Table 3:** Associations between selected independent variables and knowledge of acidic drinks (dependent variable) as causative factors for dental erosion (logistic regression)

Independent variables	Adjusted	
	OR (95 % CI)	*P*-value
Sex		
Male	1.42 (0.89 – 2.28)	0.14
Female (Ref.)		
Age		
≤35 years	**3.18 (1.33 – 7.57)**	**0.009**
≥36 years (Ref.)		
Nationality		
Yemeni	0.82 (0.41 – 1.64)	0.59
Foreign (Ref.)		
University		
Yemeni	0.80 (0.37 – 1.71)	0.57
Foreign (Ref.)		
Referral		
Yes	**0.41 (0.20 – 0.82)**	**0.012**
No (Ref.)		

Less than half the dentists and dental students (41.0 % (103/251) and 45.7 % (37/81), respectively) identified systemic diseases such as gastric reflux and eating disorders as causative factors for dental erosion. These factors were significantly more likely to be identified by male than female dental students (OR = 3.17; *P* = 0.015), while there was no significant association between sex and knowledge about these causative factors in the dentists group (*P* = 0.26). Furthermore, results from adjusted logistic regression showed that younger participants (≤35 years) were significantly more likely to identify systemic diseases, such as gastric reflux, as causative factors for dental erosion (OR = 2.7; *P* = 0.024) (Table 4). However, high sugar consumption was reported to contribute to dental erosion by 4.8 % (12/251) of dentists and 4.9 % (4/81) of dental students, while 12.0 % (30/251) of dentists and 19.8 % (16/81) of dental students thought that biting on hard objects such as pens and fingernails can cause dental erosion.

**Table 4:** Associations between selected independent variables and knowledge of systemic diseases (dependent variable) as causative factors for dental erosion (logistic regression)

Independent variables	Adjusted	
	OR (95 % CI)	*P*-value
Sex		
Male	1.08 (0.68 – 1.72)	0.73
Female (Ref.)		
Age		
≤35 years	**2.71 (1.14 – 6.46)**	**0.024**
≥36 years (Ref.)		
Nationality		
Yemeni	0.69 (0.35 – 1.35)	0.28
Foreign (Ref.)		
University		
Yemeni	0.71 (0.33 – 1.52)	0.39
Foreign (Ref.)		
Referral		
Yes	0.78 (0.41 – 1.50)	0.47
No (Ref.)		

### Clinical signs of dental erosion

Smoothening of enamel surfaces was recognized as an early clinical sign of dental erosion by 57.0 % (143/251) of dentists and 49.4 % (40/81) of dental students. During the clinical diagnostic process for dental erosion, 21.1 % (53/251) of dentists and 44.4 % (36/81) of dental students preferred to use an index to grade its severity, a difference in usage patterns that was significant (OR = 2.98; *P* ˂0.001) (Table 5). Most dentists (75.7 %, 190/251) and students (53.1 %, 43/81), however, relied instead on the general signs of dental erosion without resort to an index during the clinical diagnostic phase.

**Table 5:** Associations between participants’ answer choices with respect to various questions about dental erosion (dependent variable) and type of participant (independent variable) (unadjusted logistic regression)

Answer choice	Participant	OR (95 % CI)	*P*-value
Acidic drinks can cause dental erosion	Dental student	1.54 (0.93 – 2.55)	0.09
	Dentist (Ref.)		
Systemic diseases such as gastric reflux can cause dental erosion	Dental student	1.20 (0.73 – 2.00)	0.46
	Dentist (Ref.)		
Enamel smoothening is the early clinical sign of dental erosion	Dental student	0.73 (0.44 – 1.21)	0.23
	Dentist (Ref.)		
I prefer to use an index to grade the severity of dental erosion	Dental student	**2.98 (1.75 – 5.09)**	**<0.001**
	Dentist (Ref.)		
Asking about dietary habits will help in the diagnosis and treatment plan of the case	Dental student	0.58 (0.27 – 1.22)	0.15
	Dentist (Ref.)		
To prevent dental erosion, I advise my patients to reduce acidic drinks consumption	Dental student	**1.81 (1.08 – 3.03)**	**0.022**
	Dentist (Ref.)		
Additional preventive methods against dental erosion include the use of fluoridated tooth paste	Dental student	0.98 (0.58 – 1.65)	0.95
	Dentist (Ref.)		
I prefer to refer all cases of dental erosion to a specialist	Dental student	**2.78 (1.47 – 5.25)**	**0.002**
	Dentist (Ref.)		

Most dentists (90.8 %, 228/251) and dental students (85.2 %, 69/81) believed that asking about dietary habits during history taking could help in the diagnosis and treatment-planning phase for dental erosion. There was no significant difference between dentists and dental students regarding this practice (*P* = 0.15).

### Dental erosion preventive methods

Reduction of acidic drink consumption was advised by 47.0 % (118/251) of dentists and 61.7 % (50/81) of dental students as a preventive method against progression of dental erosion. Younger respondents (≤35 years) were significantly more likely to give this preventive advice to their patients (OR = 2.9; *P* = 0.012) (Table 6). A small percentage of dentists (5.6 %, 14/251) and dental students (2.5 %, 2/81) believed that taking muscle relaxants would have a preventive role against progression of dental erosion.

**Table 6:** Associations between selected independent variables and the recommendation of acidic drink reduction (dependent variable) as a preventive measure against dental erosion (logistic regression)

Independent variables	Adjusted	
	OR (95 % CI)	*P*-value
Sex		
Male	0.93 (0.58 – 1.47)	0.77
Female (Ref.)		
Age		
≤35 years	**2.92 (1.26 – 6.77)**	**0.012**
≥36 years (Ref.)		
Nationality		
Yemeni	0.81 (0.41 – 1.61)	0.56
Foreign (Ref.)		
University		
Yemeni	0.92 (0.43 – 1.94)	0.83
Foreign (Ref.)		
Referral		
Yes	0.70 (0.37 – 1.32)	0.28
No (Ref.)		

The use of fluoridated toothpastes was advised by 37.5 % (94/251) of dentists and 37.0 % (30/81) of dental students as an additional preventive measure against dental erosion, while 10.8 % (27/251) of dentists and 4.9 % (4/81) of dental students advised their patients to wear a night guard to prevent progression of dental erosion. A small percentage of dentists (0.8 %, 2/251) and dental students (8.6 %, 7/81) believed that stopping biting on hard objects would help in the prevention of dental erosion.

### Treatment of dental erosion

A minority of dentists (27.1 %, 68/251) and dental students (19.8 %, 16/81) preferred to treat all cases of dental erosion themselves, with 58.2 % (146/251) of dentists and 54.3 % (44/81) of dental students preferring to refer severe cases of dental erosion to a specialist. At the same time, dental students (25.9 %, 21/81) were significantly more likely to refer all cases of dental erosion to a specialist than were dentists (11.2 % (28/251)) (OR = 2.78; *P* = 0.002) (Table 5). Furthermore, participants who preferred to refer all cases of dental erosion were significantly less likely to have identified acidic drinks as a causative factor for dental erosion (OR = 0.41; *P* = 0.012) (Table 3).

## Discussion

What may be considered in-depth knowledge about causative factors, diagnosis, preventive methods, as well as approaches to early diagnosis of dental erosion was found among only half the respondents. Nevertheless, and encouragingly so, most dentists and dental students believed that enquiry about the dietary history of their patients helped in the diagnosis of dental erosion. The majority of participants reported not using an index for grading the severity of dental erosion which arguably could negatively affect the chances of early dental erosion detection, progression recording, and giving appropriate preventive advice.

Our study was conducted in Sanaa, which is the capital of Yemen, with a population of nearly 2 million, and is where most dental professionals from all governorates of the country prefer to work [20]. The number of specialists in Sanaa are very few and were excluded from the study, while general dental practitioners working in governmental and private dental clinics were included as they can generally be expected to be diagnosing and treating dental erosion cases regularly [9, 21, 22].

Final year dental students were included as a comparison group as they are likely to have more up-to-date theoretical knowledge of dental erosion compared to dentists, but who in turn have more clinical experience. This also potentially allowed us to gauge the value of continuing education courses for practising dental professionals [23]. The questionnaires were distributed to and collected from all participants in person because the postal system in Sanaa is not reliable and most dentists do not have a postal address.

To ensure a high response rate, several measures were carried out in line with a Cochrane systematic review of questionnaire investigations [24]. Thus, the questionnaire was anonymous, short, developed in English, which is the language of instruction for dental education in Yemen, and included no questions of a sensitive nature. University sponsorship of the study was mentioned together with a brief explanation about the study aims. In addition, two phone reminders after 2 and 4 weeks were given to participants who failed to return the questionnaire, and this increased the response rate [24].

The relatively high response rate achieved in this study was probably in part due to the implementation of the above-mentioned strategies. However, the higher response rate among dental students in comparison with dentists might be attributed to the fact that they are still in an academic environment, in which the importance of research might be more recognized. At the same time, anonymity of the study and lack of information about non-respondents prevented reasonable non-response analysis from being conducted. Also, dependence on anamnestic information may be considered another limitation [25].

Dental professionals are generally expected to have gained knowledge about dental erosion during their undergraduate dental education. A large number of our dentist respondents, however, seem mainly to have learned about dental erosion from their own studies. This could be attributed either to poor coverage of the subject in their undergraduate curricula, or to a low priority they may have given to the subject as students. Another possibility may be that dental erosion is still not so prevalent in Sanaa or that dental erosion is somewhat overlooked in the clinical setting. Therefore, it is recommended that dental erosion is given more importance in dental education in Sanaa by revising the dental curriculum and adding more emphasis on the subject in order to better address this oral health problem that is increasing worldwide [26].

Frequent consumption of acidic drinks and the presence of systemic diseases such as gastroesophageal reflux disease are well-established risk factors for the development of dental erosion [27–33]. In spite of this, less than half the participants recognized acidic drinks and systemic diseases as factors that can cause dental erosion. This suggests a shortfall in important knowledge among many dental professionals in Sanaa, who would be the main public providers of necessary preventive care and management of dental erosion [34, 35]. Consequently, it is likely that not all dental erosion patients will receive adequate preventive advice from their dentists. This could result in low awareness about dental erosion among patients in Sanaa as was reported in other countries [11].

Smoothening of the enamel surface is considered one of the important early clinical signs in the diagnosis and grading of severity of dental erosion [15, 36–38]. Nevertheless, only about half of the participants recognized this as a characteristic feature of dental erosion in its early stage. Therefore, it is clear that diagnosis and prevention of dental erosion in its early stages might be overlooked by a large proportion of the participants in this study. A similarly low awareness about dental erosion among dental professionals was also reported elsewhere [11].

The use of a grading index is essential for recording the presence, severity and progression of dental erosion in the clinical setting [39]. In addition, the severity of existing dental erosion cases is known to progress steadily [40]. In this study, the majority of respondents did not use such an index. Whether this was due to lack of knowledge about dental erosion indices or the practitioner’s choice is unclear from our data, but in any event it might well lead to inadequate diagnosis in the early stages, thus affecting both interventional measures and follow-up of dental erosion patients, with subsequent unpredictable progression and deterioration. This is in contrast with another study from Norway where it was found that most of the dentists used a grading index during dental erosion diagnosis [41].

Identification of dental erosion etiological factors and subsequent implementation of suitable preventive approaches frequently depend on proper dietary history taking [1]. Encouragingly, the majority of dentists and dental students in this study believed that asking about dietary habits would help in the diagnosis and decision making about treatment of dental erosive lesions. This would likely increase the chances of detecting erosive lesions and identifying their etiological factors.

A correlation between use of muscle relaxants, sugar consumption, biting on hard objects and wearing night guards, on the one hand, and dental erosion prevention, on the other, has not been reported in the literature. Surprisingly, a proportion of participants advised their erosion patients to take some muscle relaxants and to reduce sugar and acidic drink consumption to prevent dental erosion. Moreover, nearly half the respondents advised their patients to stop biting on hard objects, to wear night guards and to brush with fluoridated toothpaste. This could be due to the difficulty encountered in distinguishing between dental attrition, abrasion and erosion [18, 42]. In addition, the majority of participants in a previous study believed that sugar consumption has a role in the development of dental erosion [12] which suggests confusion between dental erosion and dental caries.

Younger respondents were more likely than older respondents to identify acidic drinks and systemic diseases as causative factors for dental erosion, as well as to advise their patients to reduce acidic drinks consumption as a preventive method. Similarly, dental students were more likely than dentists to grade the presence of erosion using an index during the clinical examination. This might be attributed to younger respondents having more current knowledge about dental erosion because of their more recent dental education. At the same time, this may indicate a need for dentists to participate in continuing education courses focused on tooth wear and especially dental erosion. Such courses have started to be available in Sanaa and dental professionals should be encouraged to participate in them actively.

Although many participants could not recognize dental erosion cases at an early stage, they seem to be able to do so in the later stages when the lesions became more apparent or closer to dentinal exposure and thus easier to detect. Indeed the results showed that a large proportion of respondents preferred to treat dental erosion cases themselves and refer only severe cases to a specialist. This may suggest that, while they lack adequate knowledge about detection of early erosion, they have enough knowledge and confidence regarding dental erosion treatment approaches, and concurs with a previous study conducted in Norway where the majority of dentists preferred to treat their dental erosion patients themselves [41].

Interestingly, dental students were more likely than dentists to refer all dental erosion cases to a specialist. This could be due to their self-perceived insufficient experience in dealing with dental erosion cases compared to their independently practising colleagues.

## Conclusions

Given the limitations and difficulties of collecting information with this type of study, the results suggest that only half of the dental professionals in Sanaa appear to have adequate knowledge about dental erosion in its early stages, its causative factors and preventive methods. In addition, self-perceived knowledge of dental erosion and approaches to its early diagnosis were insufficient among many participants. Not surprisingly, younger participants have better awareness about the causative factors of dental erosion and its preventive methods. In light of these findings, there appears to be an urgent need for improving awareness and education about dental erosion within the Yemeni dental community. This could be achieved by strengthening the subject both during dental education for dental students and in continuing education courses for dental professionals.

## Additional file

Additional file 1:
**Questionnaire on awareness and knowledge of dental erosion.** (PDF 218 KB)

## Abbreviations

DE: Dental erosion
UST: University of Science and Technology
