# Supplementary material for: Awareness and knowledge of dental erosion among Yemeni dental professionals and students
Source: BMC Oral Health. 2015 Oct 8;15:119. doi: 10.1186/s12903-015-0103-x (PMC4599026; doi:10.1186/s12903-015-0103-x)
Supplement: Additional file 1: — Questionnaire on awareness and knowledge of dental erosion. (PDF 218 KB) [file 12903_2015_103_MOESM1_ESM.pdf]

Dear Colleague, **No:………..**

Dental erosion is an increasing worldwide oral health problem which has gained considerable interest.

The objective of this research is to know the frequency of dental erosion cases in Sana'a city and the methods used by general dental practitioners to prevent dental erosion. The questionnaire is divided into two parts and simple to complete. This research is conducted by Dr. Amin Al-Ashtal- University of Bergen – Norway.

I appreciate your cooperation in advance.

A- Part I - Demographic Information:

1. Gender:
   …………Male
   …………Female

2. Age: ……………

3. Nationality: ……………………….

4. Date of graduation: ………………………...

5. University of graduation: …………………..

6. Type of dental practice:
   ……. Governmental clinical dental practice
   ……. Private clinical dental practice

B- Part II – General information about dental erosion:
<u>(Please choose only one option)</u>

7. From where have you mainly learned about dental erosion:
   ……. Dental school
   ……. Continuing educational courses
   ……. Own studies/experience/colleagues
   ……. Media (newspapers, magazines, etc)

8. I see cases of dental erosion:

……. Daily
……. Weekly
……. Monthly
……. Yearly
……. More seldom / never

9. I see cases of dental erosion more commonly in:

……. Males
……. Females
……. Equally in males and females

10. I think the prevalence of dental erosion has:

………….. Increased in comparison to the past 10 years
………….. Decreased in comparison to the past 10 years
………….. Not changed.

11. I believe that dental erosion is most frequently seen at:

……. Buccal surfaces of teeth
……. Palatal surfaces of teeth
……. Proximal surfaces of teeth
……. All teeth surfaces are affected equally

12. The most frequently affected teeth by dental erosion are:

…….Incisors
…….Premolars
…….Molars
…….All teeth are equally affected

13. I think dental erosion can be caused by:

…….. Bruxism
…….. Acidic drinks
…….. Excessive teeth brushing with hard tooth brush
…….. All of the above

14. Other causes of dental erosion include:

…….. Biting on hard objects such as nails, pens and nuts
…….. High sugar consumption
…….. Systemic diseases (e.g. eating disorders, stomach reflux)
…….. All of the above

15. The main clinical sign of **early stage** of dental erosion is:

……..Shortening of teeth
……. .Facial muscle tension
……..Smoothening of enamel surfaces
……..All of the above

16. During the diagnosis of dental erosion:

……… I depend on the clinical signs of dental erosion without using a special index to grade the severity of dental erosion.

……… I prefer to use a special index to grade the severity of dental erosion.

17. During the diagnosis of dental erosion:

……… I believe that asking about dietary habits will help in the diagnosis and treatment plan of the case.

……… I believe that asking about dietary habits will not help in the diagnosis and treatment plan of the case.

18. To prevent dental erosion I advise my patients to:

…….Reduce acidic drinks consumption
…….Take some muscle relaxant drugs
…….Reduce sugar consumption
…… All of the above

19. Additional preventive measures against dental erosion may include:
   …….Use tooth paste with fluoride
   …….Wear night guard
   …….Stop biting on hard objects
   …….All of the above

20. After diagnosis of dental erosion cases:
   ………. I prefer to treat all cases myself.
   ………. I prefer to refer all cases to a specialist.
   ………. I prefer to refer only severe cases to a specialist.

Thank you very much for your help and cooperation.

If you have any questions or inquiries, please contact Dr. Amin Al-Ashtal – Phone: 711437126. E-mail: aminashtal19@gmail.com
